# Removal of polycyclic aromatic hydrocarbons (PAHs) from water through degradable polycaprolactone electrospun membrane

**DOI:** 10.55730/1300-0527.3504

**Published:** 2022-10-04

**Authors:** Fuat TOPUZ

**Affiliations:** 1 Faculty of Engineering and Natural Sciences, Sabanci University, İstanbul, Turkey; 2 Department of Chemistry, Faculty of Science and Letters, İstanbul Technical University, İstanbul, Turkey

**Keywords:** Polycaprolactone, electrospinning, polycyclic aromatic hydrocarbons (PAHs), water remediation, PAH-DNA adducts

## Abstract

Polycyclic aromatic hydrocarbons (PAHs) are common and persistent environmental pollutants produced during the incomplete combustion of fuels. They are known for their carcinogenic and mutagenic properties. Thus, their removal from water bodies is highly crucial and has become a critical issue globally. As a solution, here an electrospun polycaprolactone (PCL) membrane with a mean fiber diameter of 2.74 ± 1.3 μm was produced by electrospinning. Water contact angle (WCA) analysis confirmed the hydrophobic nature of the PCL membrane with a WCA of 124°, which remained stable over time. Differential scanning calorimetry analysis (DSC) revealed the semicrystalline nature of the membrane with the respective melting temperature (*T*_m_) of 61.5 °C and crystallization temperature (*T*_c_) of 29.6 °C. X-ray diffraction (XRD) analysis demonstrated that the crystalline structure of the PCL membrane could be preserved after electrospinning. Scanning electron microscopy analysis revealed that the membrane could be stretched without any rupture. The PCL membrane was used to scavenge PAHs (i.e. phenanthrene and anthracene) from water; the membrane could reach equilibrium capacity in a few hours, demonstrating the rapid removal of PAHs from water. The adsorption capacities for anthracene and phenanthrene were found to be 173 ± 17 and 560 ± 51 μg/g, respectively. The adsorption data fitted well with the pseudo-first-order kinetics model for both PAH molecules. The sorption could be attributed to hydrophobic adsorption, which allowed using the PCL membrane repeatedly with ethanol exposure to get rid of the adsorbed PAHs from the membrane’s surface. The partial degradation of the fibrous membrane in water was observed due to their hydrolysis-induced bulk erosion. However, the degradation was slow for the membrane kept in the air for 3 months. Overall, the PCL membrane with inherent biocompatibility, biodegradability, and good PAH sorption performance is a promising material for water depollution from toxic PAH compounds.

## 1. Introduction

Water pollution is one of the significant issues in today’s world, which requires dealing with various kinds of toxic compounds [1–3]. Particularly, wastewater discharge is one of the major sources of common pollutants as they contain various types of chemical byproducts and reagents [4, 5]. Their release into the environment pollutes exposed areas and they quickly spread into surrounding sources, including drinking water sources. Some of these molecules, such as polycyclic aromatic hydrocarbons (PAHs), are highly toxic and even carcinogenic, and on contact, they can induce severe damage on the genome level by transforming a normal cell into a cancer cell [6].

Polycyclic aromatic hydrocarbons (PAHs) are fused aromatic rings that are present naturally in crude oil, gasoline, and coal, and they are formed during the combustion of oil, wood, tobacco, and garbage [7, 8]. PAHs are known for their potent toxic, mutagenic, and teratogenic properties. Once PAHs are taken up into the body, they undergo enzymatic reactions and produce reactive metabolites by the cytochrome P450 (CYP) enzyme. The formed reactive metabolites can later covalently bind between the nucleotides of DNA strands (i.e. PAH–DNA adduct), ultimately exerting their carcinogenic effects [9]. PAHs are globally present in almost every resource; however, being lipophilic, they are prone to accumulate on organic matter. Their presence in water sources was also detected: they exist even in drinking water sources at substantial amounts (32.45–733.10 ng/L) [10]. Although their water solubility is very low (0.001–31,000 μg/L), consistent exposure to such low concentrations can lead to the induction of carcinogenesis and tumorigenesis. In this regard, PAHs have been found in groundwater (1.0–10.0 ng/L), tap water (2.5–9.0 ng/L), surface water (10–830 ng/L), and rain-water (2.7–7.3 ng/L), and the critical PAH concentration for cancer risk was reported as 600 ng/L by the World Health Organization (WHO).

Several approaches, either specific or nonspecific, for PAH scavenging have already been reported. In the first case, PAHs and sorbent materials can interact through particular interactions, such as host-guest, ***π***-***π***, and molecular docking. The latter method relies on hydrophobic interactions between the materials and PAH compounds [11–16]. Although nonspecific strategies are considered less efficient than specific ones, previous studies revealed that both methods do not significantly differ in performance. Moreover, their response times are comparable for water remediation. The latter approach does not require any specific chemistry and functionalization step, and its production on large scales is more plausible than other complex sorbent systems.

Electrospun materials offer many benefits in environmental remediation applications because of their higher surface area per unit volume, tunable porosity, interconnected porous structure, and tunable fiber shapes (i.e. beaded, rounded, core-shell, hollow, Janus, and ribbon-like) [17, 18, 19, 20, 21]. The electrospinning technique offers handy flexible materials with a nanofibrous structure for water treatment and therefore sparked a great interest to remove toxic pollutants, such as PAHs, from water. In this regard, various synthetic and natural polymers and their combinations were electrospun into nanofibrous materials to be employed for the scavenging of PAH pollutants. In one example, cavitand-based electrospun membranes were employed for the removal of several PAHs from water [22]. The authors synthesized benzoquinoxaline-based cavitands and embedded them in polyacrylonitrile fibers. The resultant fibrous membrane showed a PAH sorption capacity of 1.32 mg/g and the sorption kinetics fitted well with the pseudo-second-order model. Another interesting electrospun membrane was developed using laccase enzyme, multicopper oxidases, and poly(lactide acid) (PLA) and PLA copolymers as carrier polymers [23]. The enzyme activity could be retained significantly after the electrospinning process (i.e. >70%) and the nanofibrous membranes exhibited very high PAH sorption capacities (0.9–2.2 mg/g). Huang et al. produced hollow Co-MOF-74 incorporated electrospun membranes with hierarchical structures for PAH removal [24]. Because of the incorporated Co-MOF-74 particles, polyvinylidene fluoride fibers showed a very high surface area (approximately 634 m^2^/g). The maximum sorption capacity (i.e. calculated through the Langmuir model) of the fibers was in the range of 160–214 mg/g for various PAHs. Uyar and colleagues reported native cyclodextrin (CD)-modified poly(ethylene terephthalate) (PET) nanofibers for the removal of phenanthrene from water, and they observed no substantial improvement in the adsorption capacity of CD-functional ones to the unmodified fibers. The same research group also reported CD-grafted electrospun cellulose acetate nanofibers for removing phenanthrene [25]. They observed nearly a 10% difference in performance between CD-functional fibers and pristine fibers. In addition to synthetic fibers, natural fibers were also employed to scavenge several PAH compounds. For example, Khan et al. used natural cotton-like fibers (kapok and cattail, both are derived from plants) for PAH removal. They have found that the PAH removal performance of kapok fibers was lower than the cattail fibers, and the sorption capacity of both fibers for phenanthrene was lower than 200 μg/g [26]. Similarly, Aspen wood fibers were used as natural fibrous sorbents for snaring PAHs with a sorption capacity range of 22.5–74 μg per gram dry wood samples [27]. Numerous sorption platforms for PAH removal can be found in the comprehensive review of Lamichhane et al. [28]. Despite the presence of many PAH sorbents, efficient, straightforward, and environmentally benign sorbent alternatives are always desired for water decontamination from PAHs.

This article describes an efficient and reusable approach for scavenging PAHs (i.e. phenanthrene and anthracene) from water using an electrospun PCL membrane. Since the membrane is solely made by PCL, it is expected to be biocompatible. Unlike many sorbents, PCL-based adsorbents do not release toxic components in depolluted water during their use. PCL is a biocompatible and biodegradable polymer that undergoes slow degradation producing caproic acid, succinic acid, valeric acid, and butyric acid [29]. PCL is a hydrophobic polymer, and its hydrophobic nature allows its use in various structures to scavenge harmful lipophilic molecules from water. In this regard, the shape and size of PCL-based sorbents play critical roles in terms of the sorption capacity and an initial response rate; for instance, nano-/micron-sized materials, such as fibers, significantly boost the sorption capacity and kinetics because of their higher surface area per unit volume. In this regard, there is a single report on the use of PCL fibers for the scavenging of anthracene, benz[a]anthracene, and benzo[a]pyrene [30]. However, these three PAHs have very poor water solubility (<22 μg/L) and the sorption performance of PCL fibers for higher water-soluble PAHs (e.g., phenanthrene) has not been reported yet. Moreover, the reusability of PCL fibers for PAH removal has not been studied either. In this study, an electrospun PCL membrane, for the first time, was used for the scavenging of phenanthrene from water. The sorption kinetics for both PAHs (i.e. phenanthrene and anthracene) were determined using kinetics models. The reusability of the membrane was tested after washing the sorbents with ethanol treatment. The stability of the PCL membrane was explored in water for 3 weeks and in the air at room temperature for 3 months.

## 2. Materials and methods

### 2.1. Materials

PCL (*M*_w_ = 80 kg/mol) and PAH molecules (i.e. anthracene and phenanthrene) were purchased from Sigma Aldrich. Chloroform (≥99.8%), methanol (≥99.8%), and ethanol (≥99.8%) were received from VWR Chemicals and used as received.

### 2.2. Production of electrospun PCL membranes

PCL pellets were dissolved in chloroform: methanol mixture (5:1) under continuous stirring. Thereafter, the solution was loaded into a 5-mL syringe fitted with blunt metallic needles. The optimum PCL concentration for bead-free fibers was observed at 15% (w/v); therefore, PCL membranes were produced at this concentration. The syringe filled with a PCL solution was placed vertically on the syringe pump. The feeding rate was set to 0.5 mL/h. A high-voltage power supply was employed to provide 15 kV. The resultant fibers were collected on a metal collector at a 10 cm distance, and the collector was covered by aluminum foil. The electrospinning process was carried out at 25 °C (±1) and 45%–50% relative humidity.

### 2.3. Methods

The fiber morphology was explored using a scanning electron microscope (Hitachi S3000 N) at 15 kV and a working distance range of 5–15 mm. Before the analysis, the samples were coated with a thin gold layer using Edwards S150B Gold Sputter Coater. The average fiber diameters (<*D*>) and their distributions were calculated by analyzing approximately 50–100 fibers using ImageJ software (National Institutes of Health, Bethesda, USA). Wide-angle X-ray diffraction (XRD) analysis was done using an Empyrean setup from PANalytical. A Cu X-ray tube (line source of 12 × 0.04 mm^2^) provided Cu K-alfa radiation with λ = 0.1542 nm. The scans were done with 2*θ*, the detector axis, moving at twice the rate of the *θ*-axis of the incident beam. Water contact angle measurements were performed using a Dataphysics OCA-30 contact angle analyzer. For static contact-angle measurement, 5 μL of water droplets were placed on the fibers, WCAs were recorded at least three different positions, and the mean value was given. The phase behavior of the PCL membrane was explored using differential scanning calorimetry (DSC, TA Q2000, UK). The PCL membrane was cooled to 0 °C and then heated up to 120°C at 10°C/min under an N_2_ atmosphere. Afterward, the sample was cooled to 0 °C at 10°C/min.

### 2.4. Sorption experiments

The sorption experiments were performed using aqueous PAH solutions. The PCL membranes (2.5 mg) were treated with the solutions of the respective PAHs. The initial concentrations of phenanthrene and anthracene were 1 mg/L and 0.044 mg/L, which were below the solubility of the respective PAHs in water. During the sorption experiments, a 3 mL sample was taken and measured with fluorescence spectroscopy. The measured solution was put back into the treated solution. The adsorption capacity for PAHs was calculated using the fluorescence intensity of the respective PAH molecules and the weight of the PCL membranes used. The reusability of the PCL membrane was tested after washing the used membrane with ethanol, which released the adsorbed PAHs from the membrane’s surface. The membrane was retreated with PAHs for 3 h. This cycle was repeated two more times, and sorption capacity was determined by measuring the fluorescence intensity of the respective PAH solution.

## 3. Results and discussion

**Figure 1:**
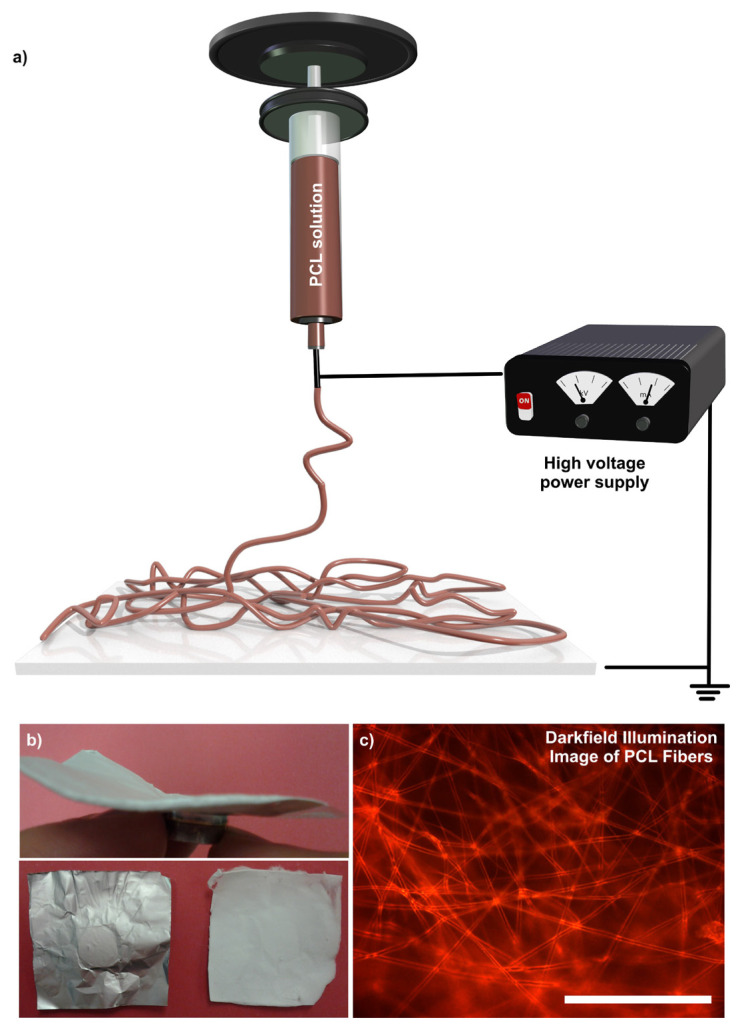
(a) A cartoon scheme of the electrospinning system employed for the production of the electrospun PCL membrane, (b) optical images of the fibers after the electrospinning process, and (c) a dark-field micrograph of the PCL membrane. Scale bar, 100 μm. *c*_PCL_= 15% (w/v) in chloroform: methanol mixture (5:1).

**Figure 2:**
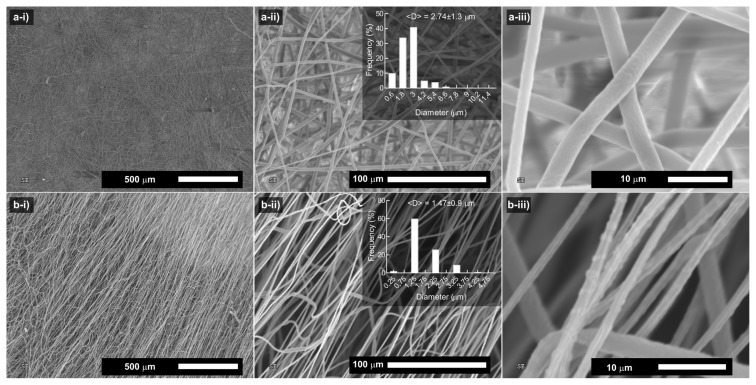
Scanning electron microscopy images of the PCL fibers (*c*_PCL_=15 % w/v) at different magnifications before (a) and after (b) stretching of the fibers. Insets show the size distribution of the respective fibers.

A PCL membrane was produced through the electrospinning of the PCL solution in chloroform: methanol mixture (5:1) (Figures 1a and 1b). The electrospun PCL fibers were collected on an aluminum foil. The membrane could easily be separated from the foil without any crack development. The dark-field illumination image of the PCL membrane, which was spun at a concentration of 15% (w/v), revealed the formation of thick fibers without any beads (Figure 1c). Scanning electron microscopy (SEM) analysis of the PCL membrane revealed the formation of microfibers. The mean diameter of the fibers was calculated to be 2.74 **±** 1.3 μm (Figure 2a). The resultant fibers are rounded and bead-free fiber structures. The high-magnification SEM image of the respective fibers demonstrated the formation of wrinkled surface texture. The fiber texture might be attributed to atmospheric pressure, which might lead to the collapse of the fast-drying skin formed on the jet surface. On the other hand, Pai et al. associated the appearance of wrinkles with the buckling of a cylindrical polymer shell under comprehensive radial stress, which resulted from the removal of solvents from the core of the jet and a lateral contraction due to the axial tensile stress [31]. The robustness of the membrane was explored through the scanning electron microscopy analysis after the membrane was subjected to stretching (Figure 2b). The stretching caused the thinning and orientation of the fibers. However, the PCL membrane could maintain its fibrous structure without any rupture, demonstrating its robustness. This can be attributed to the extension of entangled chains and their interactions with other chains over hydrophobic associations before getting deformed by an external force. With that, the mean fiber thickness decreased to 1.47 **±** 0.9 μm. PCL membrane is a mechanically robust and flexible material and does not display any crack development easily. The Young’s modulus of the PCL membrane was reported as high as 3.8 **±** 0.8 MPa, suggesting the high structural integrity of the membrane for their practical applications [32]. In industrial applications, mechanical failure of the sorbents is not desired and is the cause of sorbent replacements. Since the electrospun materials are more flexible than their nonfibrous counterparts, they will provide desired mechanical properties for their practical applications.

**Figure 3:**
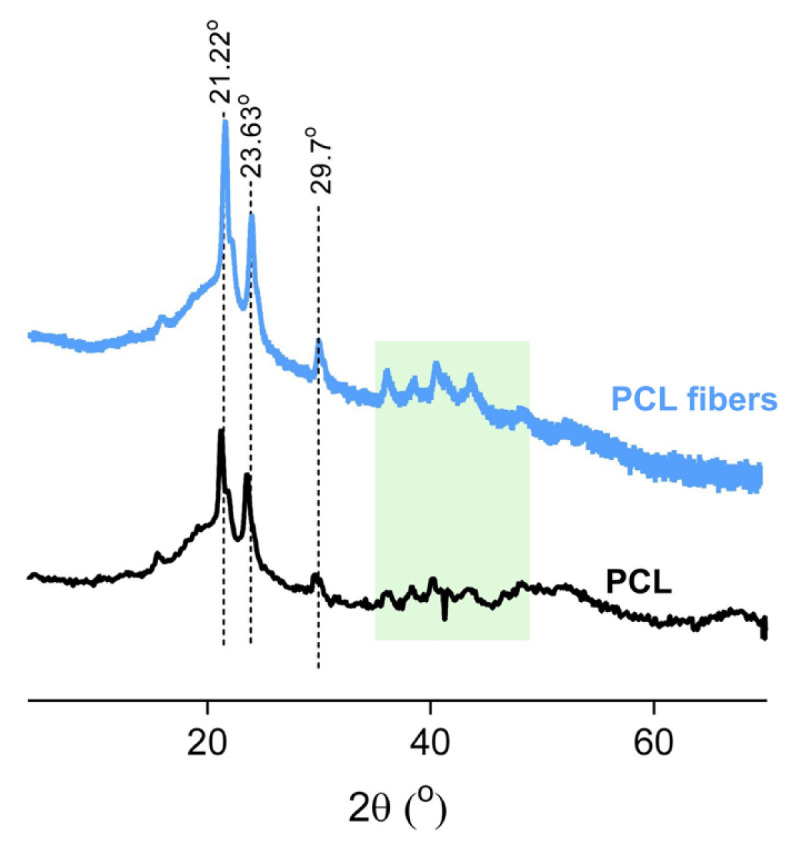
Wide-angle X-ray diffraction (XRD) patterns of the PCL membrane and film.

**Figure 4:**
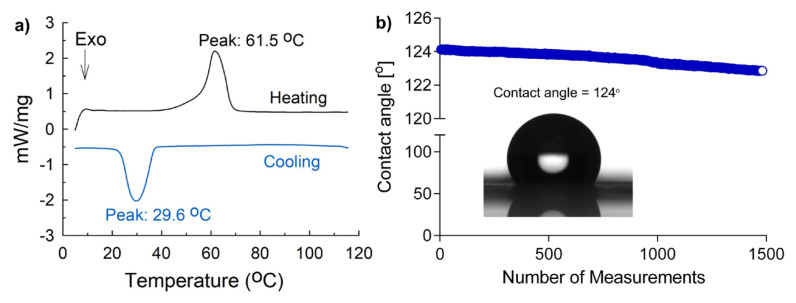
(a) DSC curves of the PCL membrane during a heating-cooling cycle. (b) Time-dependent WCA measurements of the PCL membrane. The inset photo shows a water droplet on the PCL membrane, and the initial contact angle was measured as 124°. The x-axis “number of measurements” denotes the fastest measurements possible.

The crystalline structure of the PCL membrane and film was explored through XRD analysis (Figure 3). The sharp crystalline diffraction peaks at approximately 20° and approximately 22° showed main crystalline regions with corresponding *d*-spacing values of 4.37 Å and 3.98 Å, respectively. For many polymers, electrospinning can cause a decrease in crystallinity. However, the crystalline structure of the PCL during electrospinning could be preserved, which is consistent with the findings of Olivera et al. (2003) [33]. The PCL membrane was also analyzed through the DSC measurements using a heating-cooling cycle from 0 to 120 °C. The results revealed the semicrystalline structure of the membrane (Figure 4a). The sample showed a melting temperature (*T*_m_) of 61.5 °C and crystallization temperature (*T*_c_) of 29.6 °C, which are in line with the previous reports on PCL pellets [34].The PCL is a hydrophobic molecule composed of repetitive caprolactone segments; thus, the material derived from PCL molecules should possess a hydrophobic character. The surface hydrophobicity of the PCL membrane was explored by time-dependent wettability experiments over contact angle measurements (Figure 4b). The water droplet stood as a receding sphere with the respective water contact angle of 124°. Over time, the wettability of the membrane remained almost stable.

**Figure 5:**
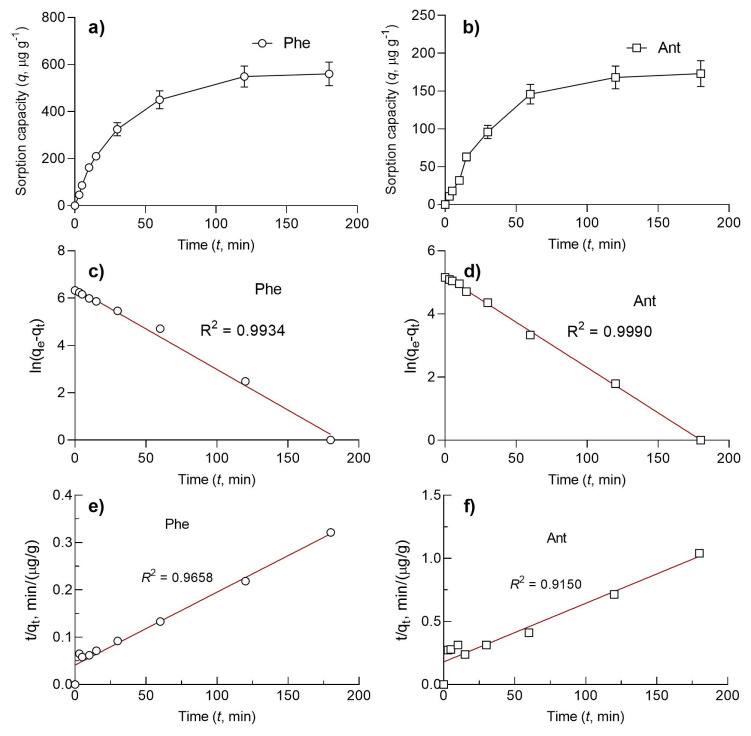
PAH sorption performance of the PCL membrane from water. Sorption capacity of the PCL membrane for (a) Phe and (b) Ant as a function of time. Pseudo-first-order kinetic model fit for (c) Phe and (d) Ant. Pseudo-second-order kinetic model fit for (e) Phe and (f) Ant.

The sorption properties of the PCL membrane were tested with PAH molecules (i.e. anthracene and phenanthrene) in water (Figure 5). From the time-dependent adsorption of the PAHs, the sorption capacity of the PCL membrane for each PAH molecule was calculated. The PCL membrane showed a higher sorption capacity for phenanthrene than anthracene. This could be ascribed to the high-water solubility of phenanthrene (i.e. 1.4 mg/L) compared to anthracene (i.e. 0.045 μg/L) at the levels they were treated with the fibers. The sorption capacities of the membrane were found to be 173 μg/g for Ant and 560 μg/g for Phen. Even though PAH sorption data fit well with both pseudofirst-order kinetic and pseudosecond-order kinetic models, experimental sorption capacity was much closer for the pseudofirst-order kinetic model with higher R^2^ values (Table 1). The PAH sorption capacity of the membrane was compared with different PAH sorbents (Table 2). The sorption capacity of the PCL membrane was higher than natural fibers but lower than the adsorbents, which rely on specific interactions, such as host-guest complexation with CDs and π–π interactions with DNA strands (Table 2). Keeping in mind the biocompatibility, biodegradability, and ease to fabricate, PCL fibers seem to be promising materials to be employed for PAH removal. The PAH sorption performance of the nanofibrous PCL membranes was compared with the performance of the previously reported PCL fibers. In this regard, Dai et al. used PCL for the removal of anthracene and found the equilibrium sorption performance as 78.6 μg/g, which is much lower than the equilibrium sorption capacity reported in this work (i.e., 173 μg/g) (30).

**Figure 6:**
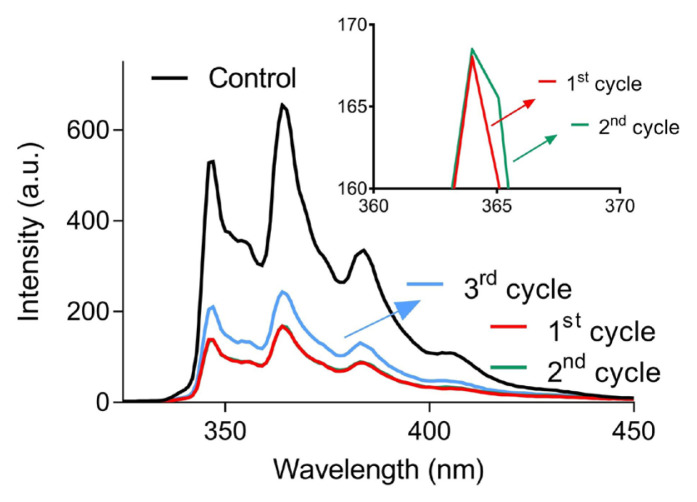
The fluorescence spectra of the phenanthrene solutions before and after three-time treatment with the PCL membrane for 3 h. The inset shows the narrow range of the respective spectra.

The PCL membrane could be reused for PAH sorption after exposure to ethanol, which released the adsorbed PAHs from the membrane surface. Afterward, the membrane retreated with a phenanthrene solution of an identical concentration. After the 3-h treatment, the intensity of the solution was comparable to the first use, while after the 3rd repetition, a slight decrease in the sorption performance was observed (Figure 6). This demonstrated the reusability of the PCL membrane for PAH removal from water bodies.

**Figure 7:**
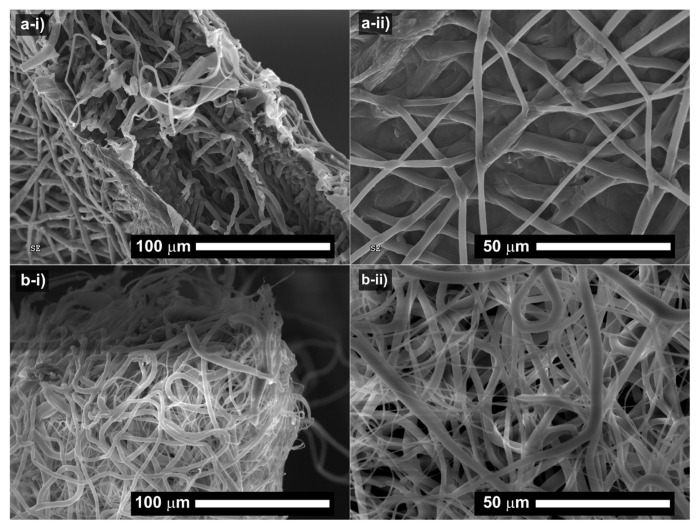
Scanning electron microscopic images of the PCL membrane at different magnifications after (a) 3-week incubation in water and (b) 3-month incubation in the air at room temperature.

One of the most intriguing characteristics of the PCL membranes is their intrinsic stability in water under mild conditions. Although PCL is a biodegradable polymer, its degradation in aqueous solutions takes a long time due to the gradual hydrolysis of ester bonds. Furthermore, its degradation is highly dependent on the incubation temperature. The PCL membrane was subjected to hydrolysis at 25 °C for 3 weeks. After 3 weeks of incubation in water, the fibers in the PCL membrane became swollen, interlinked, and degraded to some extent. Due to the hydrophobic nature of the PCL, the primary degradation pathway of PCL could be attributed to hydrolysis-induced bulk erosion, meaning the loss of material from every side. This is in line with the literature where PCL degradation occurred as the random hydrolytic breakage of the ester linkages in the PCL structure [35, 36]. With that, the swelling of fibers occurred to some extent, and the fibrous structure became obscure and exhibited a film-like structure as seen in Figure 7a. On the other hand, the degradation of the PCL membrane was much lower for the membrane left in the air for 3 months. No apparent degradation was observed in the morphology of the respective membrane (Figure 7b).

## 4. Conclusions

A facile and biocompatible approach for the scavenging of PAHs from aquatic media using an electrospun PCL membrane was reported. The PAH sorption capacities of the PCL membrane were in the range of 0.2–0.6 mg/g depending on the PAH compound. Furthermore, the PCL membrane could be reused three times with exposure to ethanol, which released the adsorbed PAH molecules from the interface but did not affect the fiber morphology. Throughout the repetitive experiments, the PCL membrane did not reveal any drastic drop in the PAH sorption capacity. The sorption results showed that the efficient binding is evident and relied on hydrophobic interactions between PAH and PCL. The sorption kinetics data fitted well with the pseudofirst-order kinetics model. The membrane could be reused after treatment with ethanol while maintaining the sorption performance above 85%. The PCL fibers could be degraded in water through mainly bulk degradation after 3 weeks of exposure, but the degradation was much slower for the fibers left in the air. Overall, the electrospun PCL membrane offers high sorption capacity for water remediation from toxic PAHs and possesses the benefits of degradability and recyclability, along with intrinsic biocompatibility.

## Figures and Tables

**Table 1:** Kinetics parameters for the adsorption of phenanthrene and anthracene by the electrospun PCL membrane.

	Experimental	Pseudofirst-order model			Pseudosecond-order model		
	*q*_exp_ (μg/g)	*q*_e_ (μg/g)	*k*_1_ (min^−1^)	*R* ^2^	*q*_e_ (μg/g)	*k*_2_ (g.μg^−1^ min^−1^)	*R* ^2^
Phe	560 ± 51	624.73	−0.00019	0.9934	649.35	0.000058	0.9658
Ant	173 ± 17	177.24	−0.00015	0.9990	215.05	0.000121	0.9150

**Table 2:** PAH sorption performances of several adsorbent systems.

Adsorbent	PAHs	Time of contact (h)	Interactions	Sorption capacity (μg g^−1^)	Reusability (%)	Reference
DNA nanogels	Ph,	3	π–π	720	N.D.	[37]
PolyCD gels	Fla, Py, Ph, Fle, An	6	Hydrophobic, inclusion-complexation	105–1250	>94 (after 2 cycles)	[38]
Kapok, cattail fibers	Ph, Fla, Fle, Nap, Acy, Ace	42–130	Hydrophobic	189 (for Ph) Kapok 193 (for Ph) Cattail	-	[26]
CD-MSN	Ph, An	3	Inclusion-complexation, hydrophobic	1650 (for Ant) 1520 (for Phe)	-	[39]
Synthetic zeolite Na-X and clinoptilolite	An	24	Hydrophobic	83–144	-	[40]
Electrospun PCL membrane	Ph, An	3	Hydrophobic	560 (for Phe) 173 (for Ant)	>85% (after 3 times)	This study

Ph: phenanthrene; Fla: fluoranthene; Py: pyrene; Fle: fluorene; An: anthracene; Ace: acenaphthene; CD: cyclodextrin; MSN: mesoporous silica nanoparticles; DNA: deoxyribonucleic acid.
